# Adaptation to prostheses among patients with major lower-limb amputations and its association with sociodemographic and clinical data

**DOI:** 10.1590/1516-3180.2014.1322572

**Published:** 2014-04-01

**Authors:** Marco Antonio Nunes, Ivo Campos-Neto, Leonardo Costa Ferraz, Camilla Andrade Lima, Tâmara Oliviera Rocha, Thaisa Fátima Rocha

**Affiliations:** I PhD. Adjunct Professor and Head of Department of Medicine, Universidade Federal de Sergipe (UFS), Aracaju, Sergipe, Brazil; II MD. Researcher and Student in the Department of Medicine, Universidade Federal de Sergipe (UFS), Aracaju, Sergipe, Brazil

**Keywords:** Amputation, Lower extremity, Prosthesis fitting, Primary health care, Health surveys, Amputação, Extremidade inferior, Ajuste de prótese, Atenção primária à saúde, Inquéritos epidemiológicos

## Abstract

**CONTEXT AND OBJECTIVE:**

Lower-limb amputation compromises patients' independence and autonomy, and therefore they should be referred for rehabilitation in order to adapt to prostheses and regain autonomy. The aim here was to assess adaptation to prostheses among patients with major lower-limb amputations and its association with sociodemographic and clinical data.

**DESIGN AND SETTING:**

This was a cross-sectional study in the city of Aracaju, Brazil.

**METHODS:**

The patients were identified by primary healthcare teams. The inclusion criterion was that these should be patients who underwent major lower-limb amputations of any etiology. Associations between sociodemographic and clinical variables and the adaptation to lower-limb prostheses were assessed.

**RESULTS:**

149 patients were examined. Adaptation to the prosthesis occurred in 40% (60/149) of them, but only 62% (37/60) were using it. Adaptation occurred more often among male patients (P = 0.017) and among those who had a higher educational level (P = 0.013), with a longer time since amputation (P = 0.049) and when the etiology was trauma (P = 0.003). The result from logistic regression analysis showed that only patients with low education (P = 0.031) were significantly associated with a lower frequency of adaptation to prostheses.

**CONCLUSION:**

It was found that patients with a low educational level became adapted to the prosthesis less frequently.

**CONTEXTO E OBJETIVO:**

A amputação de membros inferiores compromete a independência e a autonomia dos pacientes, por isso, eles devem ser encaminhados para a reabilitação para a adaptação das próteses e assim viabilizar a recuperação da autonomia. O objetivo foi avaliar a adaptação de prótese em pacientes com amputações maiores de membros inferiores e sua associação com dados sócio-demográficos e clínicos.

## Abstract (pt)

**TIPO DE ESTUDO E LOCAL:**

Estudo transversal realizado na cidade de Aracaju, Brasil.

**MÉTODO:**

Os pacientes foram identificados pelas equipes de atenção primária à saúde. O critério de inclusão foi pacientes submetidos a amputações de membros inferiores principais de todas as etiologias. Foram avaliadas as associações entre variáveis sociodemográficas e clínicas e a adaptação de próteses de membros inferiores.

**RESULTADOS:**

Foram examinados 149 pacientes. A adaptação da prótese ocorreu em 40% (60/149) deles, mas apenas 62% (37/60) a utilizavam. Adaptação ocorreu mais frequentemente em pacientes do sexo masculino (P = 0,017) e naqueles que tinham maior nível de escolaridade (P = 0,013), com maior tempo de amputação (P = 0,049) e quando a etiologia (P = 0,003) foi o trauma. O resultado da análise de regressão logística mostrou que apenas a com baixa escolaridade (P = 0,031) foi significativamente associada com uma menor frequência na adaptação de próteses.

**CONCLUSÃO:**

Verificou-se que pacientes com baixa escolaridade tiveram menor frequência de ajuste para a prótese.

## INTRODUCTION

Amputation of a limb is not only an esthetic loss: it also compromises autonomy and self-esteem, leaving the patient helpless and dependent.^1^ Therefore, autonomy and independence need to be preserved, and such patients should be encouraged to undertake self-care, recognize their limits and return to their usual activities.

These patients should be referred to a rehabilitation program. This is a fundamental part of care and essential for a good functional outcome, because it enables recovery of independence after returning to the community, such that these individuals can gain the ability to perform their usual activities as independently as possible in order to achieve optimal social participation.^2^ ^-^ ^5^ The main goal of rehabilitation is to allow integration into the community as a productive and independent member.

The rehabilitation process firstly involves careful selection of patients for use of lower-limb prostheses.^3^ The healthcare team needs to recognize the patients' clinical and social problems and understand the factors associated with successful outcomes from this process.

## OBJECTIVE

Amputation of a limb is not only an esthetic loss: it also compromises autonomy and self-esteem, leaving the patient helpless and dependent.^1^ Therefore, autonomy and independence need to be preserved, and such patients should be encouraged to undertake self-care, recognize their limits and return to their usual activities.

## METHODS

A cross-sectional study was conducted from May 12 to June 30, 2011. The research was planned in accordance with the Declaration of Helsinki and was approved by the Research Ethics Committee of Universidade Federal de Sergipe on May 6, 2011. The patients were identified by primary healthcare teams in the city of Aracaju, in Brazil and, after informed consent had been obtained from the patients, data were gathered through visits to patients' homes. Relevant data were recorded on a standardized form.

## Sample

The inclusion criteria were that the subjects should be patients who had undergone unilateral or bilateral lower-limb amputation performed at levels above the ankle joint (major amputation); and that the etiologies should relate to trauma, diabetes mellitus, infection, ischemia or cancer. The criterion for exclusion was the presence of mental impairment that precluded participation.

To calculate the sample size, it was assumed that the variable that contained the response of interest had a population prevalence of 50% adaptation to lower-limb prostheses,^6^ maximum error of estimate of 8% and significance level of 5%. Thus, the sample size was calculated as 151 individuals.

## Variables and instruments

Sociodemographic data such as age, gender, marital status and education (which was classified as low when the subjects had not completed primary education), and clinical variables such as etiology, length of time since amputation and number of associated morbid conditions, were gathered on the survey form. The subjects were also asked about occurrences of adjustment of the lower-limb prosthesis, which were defined as fitting the prosthesis for use with or without external support. Walking within the community was not differentiated from walking only at home, and patients who used the prosthesis for cosmetic purposes or for transfers were recorded as nonusers.

## Statistical analysis

We performed descriptive analyses to examine the clinical and demographic data. Continuous variables were presented as mean values and 95% confidence intervals, whereas categorical variables were presented as absolute and relative frequencies. The relationships between sociodemographic and clinical variables and adaptation to prostheses were tested through using contingency tables and calculation of Pearson's chi-square test or Fisher's exact test. Differences between the means of pairs of groups were analyzed using Student's t test. We then performed logistic regression analysis to control for confounding variables using those that showed associations with P < 0.20, with the aim of exploring the magnitude of associations between the sociodemographic and clinical variables and the results from prosthesis fitting. P values < 0.05 were considered statistically significant.

## RESULTS

Since two amputees refused to sign the informed consent statement, we interviewed 149 patients. They had undergone major lower-limb amputations at a mean age of 60.2 years (95% CI: from 57.3 to 63.2); 62% (92/149) were males, 50% (74/149) were married and 65% (97/149) reported a low educational level. The mean length of time since amputation was 76.8 months (95% CI: from 61.9 to 91.8). The amputation had been bilateral in 18% (27/149) of the patients; 45% (67/149) reported that diabetic foot was the cause of amputation; and in 26% (38/149), the procedure related to trauma. Regarding associated diseases, 23% (34/149) reported none, 53% (79/149) had diabetes mellitus and 57% (85/149) had hypertension (Table 1). Adaptation to the prosthesis had occurred in 40% (60/149) of the patients with major amputations, but only 62% (37/60) of these patients were using their prostheses daily or occasionally.

**Table 1.** Frequencies of sociodemographic and clinical variables relating to patients with major lower-limb amputations

Variable	n	%
Gender		
Female	57	38
Male	92	62
Marital status		
Married	74	50
Not married	75	50
Education		
Low level	97	65
High level	52	35
Etiology		
Diabetic foot	67	45
Trauma	38	26
Ischemia	19	13
Infections	17	11
Others	8	5
Adaptation to prosthesis		
Yes	60	40
No	89	60
Use of prosthesis		
Yes	37	62%
No	23	38%

Regarding evaluation of adaptation to the prosthesis (Table 2), this occurred among 28% (16/57) of the women and 48% (44/92) of the men (P = 0.017), and among 33% (32/97) of the patients with low education and 54% (28/52) of those with higher education (P = 0.013). Moreover, there was no influence from being married or having a partner (P = 0.285). There was also no significant differences between age groups (P = 0.146) and numbers of associated diseases (P = 0.307) among these patients (Table 3). However, those with a longer time since amputation were associated with a greater chance of successful prosthesis fit (P = 0.049).

**Table 2.** Relationship between adaptation to the prosthesis and the sociodemographic and clinical variables, among patients with major lower-limb amputations

Variable	Adaptation to prosthesis			P-value
	Yes (n %)	No (n %)	Total (n %)	
Gender				
Female	16 (28%)	41 (72%)	57 (100%)	0.017
Male	44 (48%)	48 (52%)	92 (100%)	
Education				
Low level	32 (33%)	65 (67%)	97 (100%)	0.013
High level	28 (54%)	24 (46%)	52 (100%)	
Marital status				
Married	33 (45%)	41 (55%)	74 (100%)	0.285
Not married	27 (36%)	48 (64%)	75 (100%)	
Diabetes *mellitus*				
Yes	30 (38%)	49 (62%)	79 (100%)	0.544
No	30 (43%)	40 (57%)	70 (100%)	
Hypertension				
Yes	33 (39%)	52 (61%)	85 (100%)	0.679
No	27 (42%)	37 (58%)	64 (100%)	
Diabetic foot				
Yes	25 (37%)	42 (63%)	67 (100%)	0.506
No	35 (43%)	47 (57%)	82 (100%)	
Trauma				
Yes	23 (61%)	15 (39%)	38 (100%)	0.003
No	37 (33%)	74 (67%)	111 (100%)	
Total	60 (40%)	89 (60%)	149 (100%)	

**Table 3.** Relationship between adaptation to the prosthesis, the mean age, the length of time since amputation and the number of associated diseases, among patients with major lower-limb amputations

	Adaptation to prosthesis	n	Mean	SD	P-value
Age	Yes	60	57.6	18.7	0.145
	No	89	62.0	18.0	
Length of time since amputation	Yes	60	96.3	111.7	0.049
	No	89	63.7	74.2	
Associated diseases	Yes	60	1.2	0.9	0.307
	No	89	1.4	0.9	

When the associated diseases were taken into account, although those who reported diabetes *mellitus* (P = 0.679) and hypertension (P = 0.544) showed lower frequencies of achieving prosthesis fitting, this was not statistically significant, which likewise occurred among patients who reported that diabetic foot (P = 0.506) was the cause of amputation. However, when the cause was trauma, 61% (23/38) achieved prosthesis fitting (P = 0.003).

Given these results, logistic regression analysis was applied to the data in order to assess the likelihood of an association between multiple independent and dependent variables represented by the result relating to adaptation to the prosthesis. Thus, among the variables of age, gender, low education, trauma and length of time since amputation, selection for entry into the model was based on their individual effect on the dependent variable, at a predetermined significance level, which in this case was chosen to be alpha equals 0.20. The result from this analysis is shown in Table 4. The association between the observed dependent and independent variables provided by logistic regression analysis showed that only the variable of low education (P = 0.031) was significant (Table 4).

**Table 4.** Result from logistic regression analysis

Variable	Estimate	Standard error	W test	P-value
Age	-0.0071	0.0120	0.3570	0.5502
Gender	0.5327	0.3958	1.8114	0.1783
Education	0.8153	0.3782	4.6489	0.0311
Trauma	-0.9012	0.5097	3.1263	0.0770
Length of time since amputation	-0.0019	0.0021	0.7689	0.3806

## DISCUSSION

Our results regarding adaptation to prostheses were very similar to those obtained by other authors,^3^ ^,^ ^6^ ^,^ ^7^ although only 62% of our patients were using their prosthesis daily or occasionally and thus a significant number of the amputees were unable to regain the ability to walk even with rehabilitation. Despite technological advances in scientific knowledge, the results regarding prosthesis use have varied,6,8,9 and therefore the predictors for prosthetic rehabilitation remain unknown. The purpose of using a prosthesis is to compensate for the functional loss,10 because although locomotion with a wheelchair can be a good alternative due to its energy consumption, mobility becomes very limited. In addition, prostheses have an important social and cosmetic influence and help to avoid disturbances to body image. Thus, they have a significant influence on psychosocial adaptation to amputation11 and on performance in activities of daily living.^12^

Therefore, since social restrictions have an impact on patients' lives, rehabilitation should be an ongoing, individualized and planned process, from before the operation until the definitive prosthesis is inserted, in order to allow patients to recover normal life and perform all their basic activities. However, dissatisfaction with and low utilization of physiotherapy and occupational therapy services have been reported, which may be related to cost, difficulty of access or lack of availability of services and their potential benefits.^13^

We observed that patients with low education less frequently adapted to the prosthesis. One explanation for this finding may be what has been reported previously, i.e. that those with higher educational levels use rehabilitative care more often.^14^ Moreover, not every amputee is a candidate for a prosthesis and therefore it is necessary to know how to recognize their problems, including social and economic issues. In addition, just as in our study, females have been associated with a worse outcome from the rehabilitation process.^15^

Longer time since amputation was also associated with a greater chance of success. However, delays in referral to rehabilitation services are sometimes unavoidable, and this can lead to development of joint contractures and local complications, thereby making adaptation to the prosthesis more difficult.^16^ Factors relating to the prosthesis have been most correlated with the outcome from rehabilitation.^17^

Our results revealed that individuals who reported diabetes *mellitus* and hypertension showed a slight tendency towards a lower frequency of adaptation to the prosthesis, although this was not statistically significant. Nonetheless, it has been shown that associated diseases compromise amputees' independence.^3^ ^,^ ^18^ Moreover, when amputation occurs among elderly people, at a level above the knee or bilaterally, those who previously could not walk and had multiple associated morbid conditions are found to fail to adapt to the prosthesis or present a lower chance of using it.^6^ ^,^ ^8^ ^,^ ^19^ We also observed that when the etiology of amputation was trauma, 61% were able to adapt to the prosthesis, perhaps because most of these patients were young and had few diseases. In addition to hypertension and diabetes, peripheral arterial disease is also associated more frequently with non-traumatic amputations.^12^

One of the limitations of this study was that no inference could be made in relation to causality, because the research design was cross-sectional. Moreover, the morbid conditions were self-reported. However, despite the number of studies already available, many questions still remain to be answered in this field and prospective studies are needed on the predictors for a proper fit and for achievement of walking ability among patients, in order to guide appropriate indications for the rehabilitation process. 

Discussion on the evolution of patients undergoing lower-limb amputation is necessary because this should not be considered to be the end of the therapeutic procedure, but a new stage. Thus, these patients need help to cope with limb loss and reorganize their lives in view of the new reality. For this reason, surgeons should provide guidance for patients, including in relation to period of rehabilitation, thus establishing a proper physician-patient relationship. However, this may become a problem when the physician has not had adequate preparation, and therefore simply operating these patients is not enough.

## CONCLUSION

Therefore, considering the importance of orientation among healthcare professionals towards better care for these patients and appropriate referral to rehabilitation services, we found that the prevalence of adaptation to lower-limb prostheses was 38% and that patients with a low educational level less frequently adapted to the prosthesis. Since such adaptation and regaining the ability to walk are the main objectives of the rehabilitation process, knowledge of these factors can help the healthcare team to provide better care for patients with such characteristics. In this manner, patients can regain function and quality of life after amputation, through encouragement of better self-care, lower dependence, greater range of social interactions and less isolation, and also through promotion of preventive actions.
